# Does peer teaching improve academic results and competencies during medical school? A mixed methods study

**DOI:** 10.1186/s12909-022-03507-3

**Published:** 2022-06-04

**Authors:** Marijke Avonts, Nele R. Michels, Katrien Bombeke, Niel Hens, Samuel Coenen, Olivier M. Vanderveken, Benedicte Y. De Winter

**Affiliations:** 1 Skills lab, Faculty of Medicine and Health Sciences, Campus Drie Eiken, University of Antwerp, Universiteitsplein 1, 2610 Antwerp, Belgium; 2 Department of Family Medicine and Population Health, Faculty of Medicine and Health Sciences, University of Antwerp, Antwerp, Belgium; 3 Department of Epidemiology and Social Medicine (ESOC), Faculty of Medicine and Health Sciences, University of Antwerp, Antwerp, Belgium; 4 Vaccine & Infectious Disease Institute (VAXINFECTIO), Faculty of Medicine and Health Sciences, University of Antwerp, Antwerp, Belgium; 5 I-Biostat, Data Science Institute, Hasselt University, Hasselt, Belgium; 6 Otolaryngology and Head and Neck Surgery, Antwerp University Hospital, Antwerp, Belgium; 7 Laboratory of Experimental Medicine and Pediatrics, University of Antwerp, Antwerp, Belgium

**Keywords:** Peer assisted learning, Peer teaching, Clinical skills, Competencies, CanMEDS

## Abstract

**Background:**

This study investigates the impact of Peer-Assisted Learning (PAL) in clinical skills on peer teachers’ academic scores and competencies; however, controversy remains on this topic, and concrete evidence on its impact lacking.

**Methods:**

We performed a mixed methods study combining a retrospective cohort study with a modified Delphi survey. Peer teachers and Skills Lab faculty members participated in this study. A validated questionnaire, the CanMEDS Competency Based Inventory (CCBI), and group interviews were used to assess the outcomes of PAL. Our results were also triangulated with literature data.

**Results:**

In 3 consecutive cohorts of medical students (*n* = 311), 78 participated in PAL. Peer teachers obtained higher scores from the start of the study, at different timepoints in medical school, and on their final scores compared to all other students. Interestingly their progress followed the same path and magnitude as other well-performing students. However, based on our findings from a modified Delphi survey (CCBI interviews) and a literature review, we found further supporting evidence for a positive impact of PAL on the competencies of physical skills (medical expert), teamwork and leadership (collaborator), lifelong learning (scholar), and for admitting uncertainty/limits (professional) within the CanMEDS roles.

**Conclusions:**

We conclude that higher achieving students are more likely to volunteer for a peer tutoring program; however this does not significantly augment their academic scores as compared to above well-performing non-teaching fellow students. Importantly, our modified Delphi survey indicated which CanMEDS roles were positively impacted by PAL: medical expert, collaborator, scholar and professional.

**Supplementary Information:**

The online version contains supplementary material available at 10.1186/s12909-022-03507-3.

## Background

Peer-assisted learning (PAL) is a promising concept of involving medical students in a teaching role at an early stage of their training; a role they will also undertake later as a medical doctor [1] and resident [2]. According to a recent review by Hermann-Werner et al. [3], allowing medical students to take on a teaching role improves their own learning process [2, 4, 5]. PAL can include more advanced students teaching less advanced students (“near-peer”) as well as students teaching fellow students within the same educational level and academic year (“peer”). PAL benefits the (near-) peer teacher in 2 different ways: they become better learners by understanding learning principles and it supports their development by boosting their self-confidence and organizing peer teaching sessions [3]. However, it remains unclear which exact competencies that peer teachers achieve during this process. The impact on different competencies is rarely assessed using a validated model such as the CanMEDS framework [6]. Most studies have been based on self-reported questionnaires from (near-) peer teachers themselves.

Furthermore, the impact on academic capabilities of the medical students’ clinical skills serving as peer teachers in clinical skills is still a topic of debate [3]. Iwata et al. concluded that students who acted as (near-) peer teachers performed better in final-year examinations, but this difference was attributed to their better scores in their 4th year of medical school [7]. In the study by Knobe et al., peer teachers showed better results at Objective Structured Clinical Examination (OSCE) on ultrasound examination [8]. Nestel et al. revealed no changes in patient-centered interviewing skills after PAL [9]. However, these results could have been affected by a selection bias as it has been suggested in the literature that high achieving students self-select as (near-) peer teachers [7].

In our study we aimed to investigate 2 aspects of PAL. First, we explored the potential effects of a selection bias. We specifically designed our study to consider the academic level of the participating students. The students’ academic level was noted at the start of the study, and we followed the students’ academic trajectory longitudinally throughout medical school. Second, we aimed to systematically determine the impact of PAL for medical skills on all CanMEDS roles, which we assessed using varied approaches.

## Methods

### Context

**Fig. 1:**
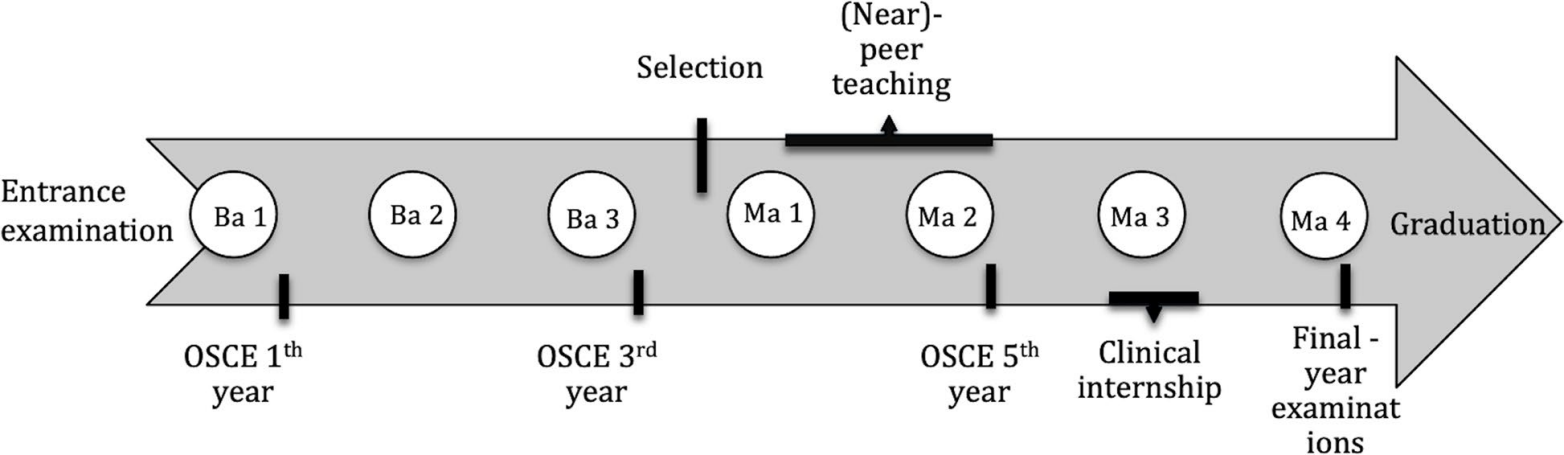
Timeline of the 7 year medical curriculum at University of Antwerp. Ba: Bachelor; Ma: Master; OSCE: Objective Structured Clinical Examination

During medical school (a curriculum of 6 years (7 until 2018)) students at the University of Antwerp can apply for a voluntary (near-)peer teaching program in the Skills Lab during their 4th and/or 5th year, which means 1 or 2 years before starting their clinical internship but after obtaining their Bachelor’s degree (Fig. 1). Our program follows the classification of Olaussen et al., in terms of near-peer and peer teaching^1^ [10].

The selection of the students was based on a completed application form, in addition to a curriculum vitae and a cover letter explaining their motivation to become a peer-teacher. When the peer teachers were selected (by 2 faculty members from the Skills Lab), they would choose 1 or 2 topics out of a list of all OSCE stations to teach: heart and lung, abdomen, basic life support (BLS) and first aid, suturing, intramuscular (IM) injection and drawing blood, gynecology, musculoskeletal examination, neurology, eye examination and ear, nose, throat (ENT) examination, taping, and examination of a neonate. Subsequently, the selected students received training of approximately 2 hours per teaching topic from an experienced staff member. During this training, they practiced the necessary clinical, physical and technical skills and received advice on how to teach and provide feedback (didactic skills).

In this peer teaching program students teach the different physical examination skills to their fellow students (3rd to 5th year) and coach them during additional practice sessions. This provides added training sessions for students, in addition to the official skills training. It also supports students in mastering the necessary skills and provides an ideal preparation for the OSCE (Objective Structured Clinical Examination) that takes place at the end of both the 3rd and 5th years of medical school. Students need to succeed at these high stakes OSCEs before they can enter a Master’s program or a fulltime clinical internship year.

During the peer skills sessions, 5 to 8 peer teachers present for up to 30 to 50 students. Each peer teacher provides 3 to 5 sessions a year. For each topic a coordinator is appointed for the group of peer teachers. These coordinators are supervised by the faculty staff member(s) from the Skills Lab to whom they report.

### Study design

We performed a mixed methods study combining a retrospective cohort study to assess the effects of PAL on the students’ academic scores throughout their entire medical school career. We used a modified Delphi survey to triangulate information from peer teachers and Skills Lab faculty members with data from the literature to systematically investigate which CanMEDS competencies were affected by PAL.

#### Retrospective longitudinal cohort study

We analyzed 3 consecutive cohorts of peer teachers between 2009 and 2013. Only students who had completed their full medical school training at the University of Antwerp were included. We included examinations and assignments from the Bachelor’s years, before the start of the peer teaching program, and from the Master’s years, after the peer teaching program reflecting different skills and competencies (Table 1). During the entire curriculum students learn medical-technical (physical) and communication skills in a ‘clinical line’. For this clinical line students need to develop a portfolio, representing a combination of self-reflections, case reports, and personal development plans. At the end of their 1st, 3rd and 5th year they need to pass a high-stakes OSCE. For this study, we collected: (a) the ‘clinical line’ scores (1st to 6th year, portfolio), (b) all the OSCE scores, (c) scores of the students’ high stakes internship portfolio (6th year workplace portfolio, combining different types of assignments), (d) the case-based clinical examination (7th year, 1 live patient and 3 paper cases assessed by a jury of 4 faculty members), and (e) the final multiple choice questionnaire (MCQ) test (7th year). To be complete we also used (f) the students’ final Bachelor’s and Master’s scores. We assumed that all these scores represented the students’ objective academic outcomes.

**Table 1:** Overview of included examinations and assignments, and the CanMEDS roles they assess

Examinations and assignments	Skills & CanMEDS roles
(a) Clinical line portfolio	Combination of medical expert, communicator, leader, scholar, and professional roles
(b) OSCE	Medical-technical and communication skills
(c) Internship portfolio	Combination of medical expert, communicator, leader, scholar and professional roles [11]
(d) Clinical examination	Clinical reasoning and medical decision making
(e) Final MCQ test	Medical knowledge
(f) Final Bachelor/Master score	Global academic achievements

#### Modified Delphi study

We used the validated CanMEDS Competency Based Inventory (CCBI) (URL: https://bmcmededuc.biomedcentral.com/articles/10.1186/1472-6920-12-86/tables/3) [12] to systematically assess which competencies were obtained during our PAL program. In our modified Delphi survey, the CCBI was sent by e-mail to faculty members of the Skills Lab who participated in this research (*n* = 5) and to all peer teachers (*n* = 45) who participated in the PAL program during the academic year 2015–2016. This is a different cohort of students than the retrospective cohort study. They were asked to mark on a yes/no scale which competencies they believed they obtained through the peer teaching program. All answers were anonymously summarized in a matrix. In the next phase, all respondents were invited for a moderated face-to-face interview (one with the faculty members, one with the peer teachers) where the matrix was used to reach a consensus within the groups.

This Delphi-method was triangulated with a literature study based on a PubMed search with updated e-mail alerts until September 2021. The search terms used in the strategy included:

- ‘(near-peer teaching OR peer teaching OR peer tutoring OR peer tutor OR peer assisted learning) AND (academic performance)’
- ‘(peer tutoring) AND (education [MeSH Terms])’
- ‘peer assisted learning’ AND ‘medical education’
- ‘student as teacher [Title/Abstract]’
- ‘resident as teacher [Title/Abstract]’,
- ‘near-peer teaching’.

There was no time and language limit. The list of articles obtained was extended with relevant references of the selected papers according to the snowball effect.^2^

We selected relevant articles based on their title and abstracts. One author (MA) read the full articles with a focus on which medical student competencies could be improved by PAL in medical students. Only reviews were included. We compared the competencies described in the articles with the formulation of the CanMEDS competencies, searching for a match in the description of that specific competency.

### Statistical analyses

#### Retrospective cohort study

Step 1: the outcomes between the achievements of peer teachers and their fellow students at baseline (year 3) were first compared using a t-test. Using this approach, the potential selection bias was confirmed and therefore the statistical analyses were completed using step 2.

Step 2: a repeated measures ANOVA analysis using linear mixed model methodology was performed to investigate the interaction of being a peer teacher. The most parsimonious model based on AIC (Akaike information criterion) was retained.

#### Modified Delphi study

To investigate whether there were any differences between peer teachers who participated in the modified Delphi survey and those who did not, a chi square analysis was performed comparing gender, OSCE score of the 3rd year, and the final Bachelor’s score.

Effect estimates were reported with 95% confidence intervals and *p*-values. A *p*-value of less than 0.05 was considered statistically significant. Statistical analyses were performed using SPSS version 24 and R version 3.3.2.

## Results

### Retrospective cohort study

#### Step1

**Fig. 2:**
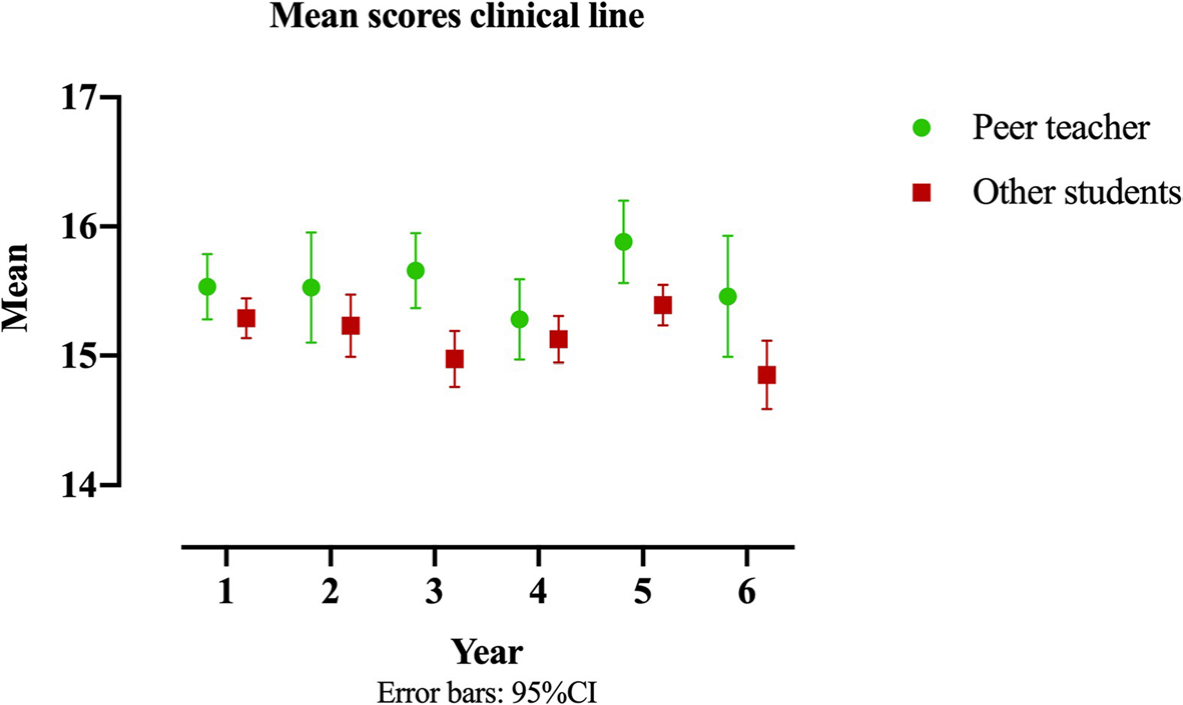
Mean scores for clinical line for 1st year until 6th year of medical school. Peer teachers are denoted in green and other students in red. Peer teachers’ scores were significantly higher in Year 3, Year 5 and Year 6

We included a total of 311 students (59% female) of which 78 students (68% female) participated as peer teachers in the PAL program of our clinical Skills Lab. Peer teachers scored significantly higher compared to their fellow students on their Bachelor’s score, internship portfolio, and final Master’s score (Table 2). The mean scores of the ‘clinical line’ from year 1 until year 6 are shown in Fig. 2. Peer teachers scored significantly higher on ‘clinical line 3^rd^ year’ and ‘clinical line 5^th^ year’.

**Table 2:** The achievements of peer teachers and their fellow students

			Peer teacher		Fellow students		MD	95% CI	
Year	Score	N	Mean	SE	Mean	SE		Lower	Upper
3rd	Bachelor^a^	311	72.90	0.86	70.88	0.48	2.02	3.91	0.13
6th	Internship portfolio^b^	307^c^	15.46	0.24	14.85	0.13	0.61	1.13	0.07
7th	Final clinical examination^b^	305^c^	14.21	0.35	14.21	0.17	0.002	0.77	−0.763
7th	Final MCQ test^b^	304^c^	14.70	0.21	14.40	0.13	0.31	0.79	−0.18
7th	Master^a^	303^c^	77.74	0.60	76.35	0.33	1.39	2.71	0.07

Student’s t-test, *MD* Mean Difference, *SE* Standard Error)

^a^Score out of 100

^b^Score out of 20

^c^Not all students graduated from medical school (*n* = 8)

#### Step 2

The final mixed model (Table 3) demonstrated that peer teachers’ scores were 0.413 (95% CI: 0.107–0.719) out of 20 higher in all years. The effect of being a peer teacher remained constant over the years, which suggests that being a peer teacher had no influence on the students’ scores throughout the clinical line.

**Table 3:** Final model estimates, SE (Standard Error) and t-value

Fixed effects	Estimate	SE	t value
Year 1	15.247	0.098	155.303
Year 2	15.203	0.098	154.850
Year 3	15.044	0.098	153.234
Year 4	15.062	0.098	153.418
Year 5	15.411	0.098	156.967
Year 6	14.905	0.099	151.314
(near-) peer teacher	0.413	0.156	2.652

In an exploratory analysis we compared the improvement of the peer teachers to fellow students with matching scores and recalculated this effect in these 2 groups of well performing students. There was no significant difference (*p*-value 0.73) between both groups.

### Modified Delphi study

All 5 faculty members (Skills Lab lecturers) participated in the CCBI-based survey and the face-to-face interview. Of the 45 peer teachers asked to participate by e-mail, 24 participants returned the completed CCBI-based survey, of which 16 were willing to participate in the face-to-face interview. Ultimately, 10 peer teachers attended the interview. We compared the peer teachers who participated in both parts to the other peer teachers based on gender, their OSCE 3rd year score and their final Bachelor’s score. There were no statistical differences in age or academic scores between these 2 groups.

**Fig. 3:**
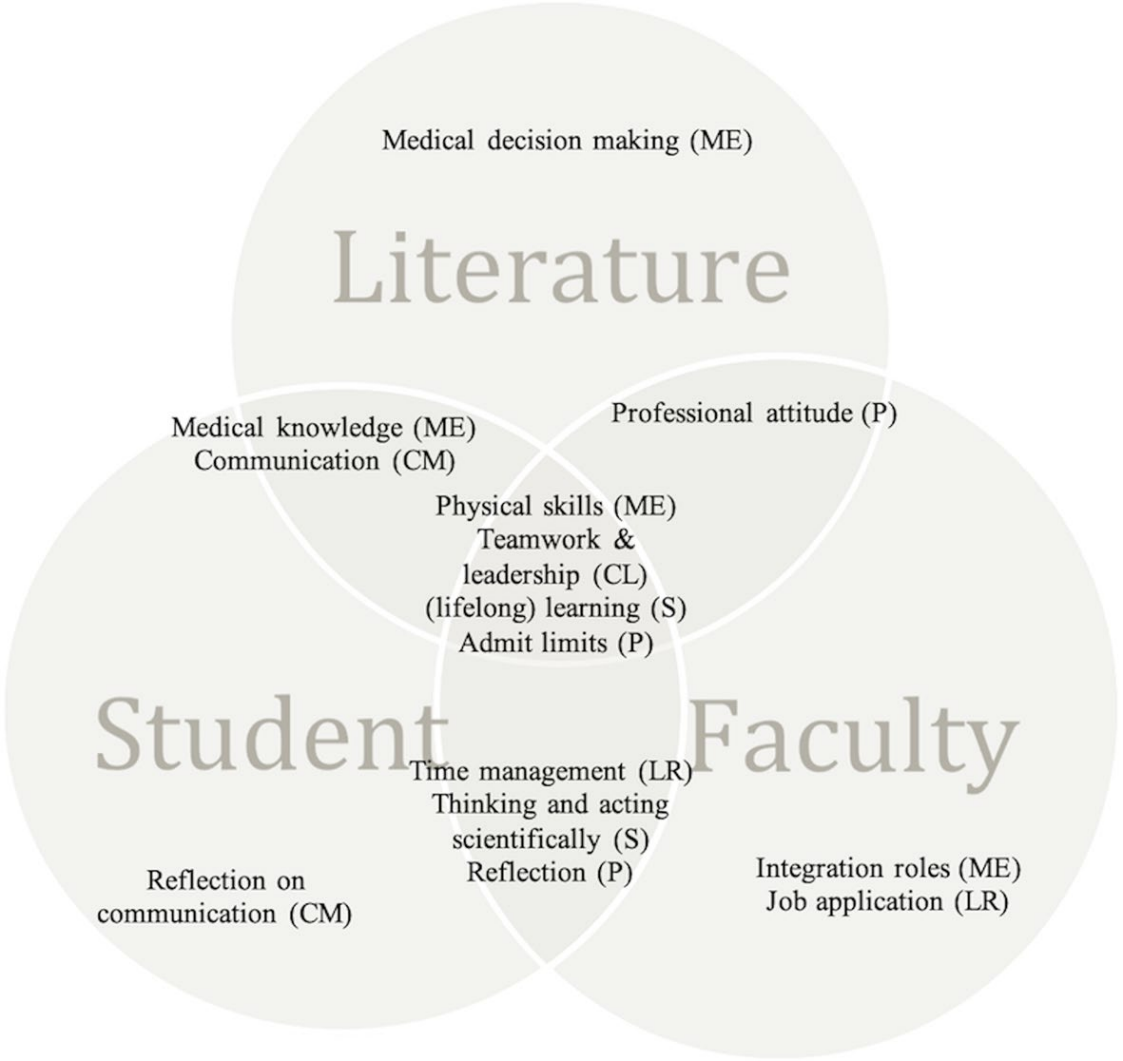
Venn diagram of competencies obtained by peer teaching based on the literature, the student- (peer teacher) and faculty member CCBI-survey and interviews. CanMEDS roles: ME = medical expert, CM = communicator, CL = collaborator, LR = leader, HE = health advocate, S = scholar, P = professional

Based on the CCBI-survey, faculty members stated that peer teachers obtained competencies mainly in the role of medical expert (e.g., medical skills, integration), collaborator (teamwork, taking responsibility, coping with conflicts, respecting the opinions of others) and scholar (performs searches, personal learning plan, stimulates training of students, lifelong learning). Faculty members also noted the peer teachers attained the roles of leader (reflects on self-care, professional time management, administrative and organizational tasks, insight into job applications) and professional (reflection, professional attitude, recognizes own limits) (Fig. 3).

Peer teachers stressed that they mainly acquired competencies on the collaborator (teamwork, taking responsibility, respecting the opinions of others) and scholar role (poses relevant questions, personal learning plan). They also seemed to acquire some competencies in the medical expert (applying knowledge, clinical skills), communication (communicates and reflects on own communication skills) and professional roles (reflection, recognizes own limits) (Fig. 3).

Based on the literature search, 7 reviews were included that specifically focused on peer teachers’ competencies obtained by PAL. These reviews described competencies as medical expert (clinical (reasoning) skills [5, 13], knowledge improvement [14]), communicator (more effective communication skills [2, 5]), scholar (having a better understanding of teaching and learning principles [2–4, 13–15]), professional (professional attitude [15]), and collaborator (developed leadership qualities, learn to admit their uncertainty [3, 13, 15]). (Fig. 3).

## Discussion

In this study we found evidence that peer teachers improve their academic scores; however this is due to a selection bias as better-performing students self-select for the peer teaching program. However, the PAL program clearly shows beneficial effects. The peer teachers improve their competencies in the CanMEDS roles medical expert, collaborator, scholar and professional. This finding was determined via varied, quantitative and qualitative approaches, and based on the literature, the students self-assessment, and their faculty supervisors.

In the first part of our study, we compared academic scores of peer teachers and their fellow students before and after the PAL project. The former outperformed the latter on the clinical line of the 3rd year, the final Bachelor’s scores (before PAL), the clinical line of the 5th year, the scores of the internship portfolio and the final Master’s scores (after PAL). Our analyses revealed that this performance was not due to their participation in PAL per se*,* but rather reflected the presence of a selection bias - the better performing students were the students who applied for the peer teaching program. The finding was corroborated by comparing the peer-teachers to a group of equally well-performing fellow students, which eliminated the differences between peer-teaching students and non-peer teaching students.

Some other studies have described that peer teachers obtained higher scores after teaching theoretical subjects such as basic sciences [16] or a surgical seminar [17]. Iwata et al. focused on clinical skills [7] but included only Year 4 results as background academic ability. Our results differentiate from this outcome and demonstrate how peer teachers follow the same path as other well-performing students. We were able to include the entire medical career of the students, which strengthens our findings.

The data do not show an increase in outcomes after the PAL program. The reason for this lack of outcomes may be due to circumstantial conditions. For example, peer teachers coach their fellow students in the basic clinical skills during rehearsal sessions in a relaxed atmosphere. This setting does not guarantee better results during the stressful examination conditions of an OSCE or other examinations. Moreover, the peer teachers already perform very well, and as a result there might only be minimal room for improvement, which is often referred to as the “ceiling” effect [18, 19].

In the second part of our study, we explored whether PAL influenced the CanMEDS competencies through an extensive literature search, and a validated peer teacher and faculty inventory (CCBI). There was agreement between the literature, the faculty members, and the peer teachers in that peer teachers not only obtained physical skills (medical expert), but other competencies such as functioning as a team member or a leader (collaborator), a lifelong learning attitude (scholar), and admitting uncertainty and limits (professional) (Fig. 3). This variety of competencies is presumed to be very important for functioning adequately in the future role as medical doctors. Moreover, students gain the important skill of teaching, which is invaluable in medicine, both for teaching patients and for teaching future medical trainees. Being able to admit uncertainty is one of the positive attributes of clinical teachers as role models [20], or as Epstein suggests errors in medicine may result from over certainty [21]. Learning to function in a team as a member and as a leader prepares future doctors for “team leadership”, which is becoming more prevalent within healthcare education [22].

Alvarez [23] interviewed anatomy peer teachers to explore which competencies they had developed. Homberg [24] analyzed which competencies were present in the didactic qualification program for peer skills teachers using input from peer teachers and training coordinators. Both studies used the CanMEDS framework and concluded that all roles, with exception for health advocate, were present. Our study contributed to the literature by systematically analyzing which competencies peer teachers in a Skills Lab obtained by triangulating input from peer teachers, faculty members and recent literature.

However, why this effect did not relate to higher achievements in the final examinations (6th and 7th year), needs to be studied in more detail and needs to be related to the spectrum of competencies tested during these examinations. As Umapathi [25] suggested, PAL may help to facilitate the attainment of higher levels in Kirkpatrick’s hierarchy of learning, for example long-term behavioral change, which is not measurable in examinations. He further argued that PAL is ideal for transmission of values and competencies such as communication, empathy and interprofessional liaison, areas which medical schools typically struggle to adequately address.

Smith et al. suggested that training physicians as teachers can reinforce their clinical skills and may have an effect on their development as physicians [26]. Whether obtaining a higher level of competencies has an impact on becoming a better doctor, enabling better patient-doctor relationships and increased “compliance/adherence” warrants further investigation.

We need to emphasize the strengths and limitations of our study. First, we only included certain assignments, and we did so using a retrospective study. Next, we did not include a control group in the survey examining which competencies were affected by PAL. In addition, there was no correction for previous teaching experience. On the other hand, we were able to analyze data from the same students during 7 years of medical school.

Future research regarding PAL should not only focus on assessment of competencies in the scholar, collaborator, leader and professional roles but should also take into account the selection bias during the experimental design. In the future, it would be important to investigate whether physicians who were peer teachers during their medical school training are better performing medical doctors.

## Conclusion

We conclude that better performing medical students are more likely to volunteer for a peer teaching program. Their scores follow the same trajectory as those of above-average performing non-peer teaching fellow students, without any evidence for an additional benefit of PAL. Nevertheless, it appears that they do improve their competencies on the CanMEDS roles of medical expert, scholar, collaborator, and professional.

## Supplementary Information

**Additional file 1: Figure S1.** Flowchart of article search process.

## Data Availability

The datasets used during the current study are available from the corresponding author on reasonable request.

## Footnotes

1 For the readability of this paper, from now on we will use the terms peer-teaching and peer-teacher, to refer to both near-peer- and peer teaching/teacher.

2 The snowball effect is tracking down references in the selected papers to find other relevant articles on your subject.
