# Supplementary material for: Does peer teaching improve academic results and competencies during medical school? A mixed methods study
Source: BMC Med Educ. 2022 Jun 4;22:431. doi: 10.1186/s12909-022-03507-3 (PMC9167556; doi:10.1186/s12909-022-03507-3)
Supplement: Supplementary file 1 — Additional file 1: Figure S1. Flowchart of article search process. [file 12909_2022_3507_MOESM1_ESM.docx]

**Does peer teaching improve academic results and competencies during medical school? A mixed methods study.**

Marijke Avonts , Nele R Michels, Katrien Bombeke, Niel Hens, Samuel Coenen , Olivier M Vanderveken, Benedicte Y De Winter

Additional File 1

Additional records identified through other sources
(n = 55)

Records identified through database searching
(n = 194 )

Records after duplicates removed

= records screened
(n = 178)

Full-text articles assessed for eligibility
(n = 54)

Studies included in synthesis
(n = 7)

Figure S1: Flowchart of article search process
